# FR-BINN: Biologically Informed Neural Networks for Enhanced Biomarker Discovery and Pathway Analysis

**DOI:** 10.3390/ijms26146670

**Published:** 2025-07-11

**Authors:** Yangkun Cao, Chaoyi Yin, Xinsen Zhou, Yonghe Zhao

**Affiliations:** School of Artificial Intelligence, Jilin University, Changchun 130012, China; yincy22@mails.jlu.edu.cn (C.Y.); xszhou24@mails.jlu.edu.cn (X.Z.)

**Keywords:** biologically informed neural network, chronic inflammation, explainable artificial intelligence, biomarker

## Abstract

Chronic inflammation plays a pivotal role in human health, with certain inflammatory conditions significantly increasing the risk of cancer, while others do not. However, the molecular mechanisms underlying this divergent risk remain poorly understood. In this study, we propose FR-BINN, a biologically informed neural network framework for disease prediction and interpretability. Incorporating Fenton reaction (FR)-related biological priors and leveraging multiple interpretability methods, FR-BINN identifies key genes driving cancer-prone and non-cancer-prone chronic inflammatory diseases. The experimental results demonstrate that FR-BINN achieves superior classification performance while offering biologically interpretable insights. Moreover, attribution results derived from different explainable techniques show high consistency, and intra-method results exhibit distinct patterns across disease categories. We further combine large language models with feature attributions to identify candidate biomarkers, and independent datasets confirm the robustness of these findings. Notably, genes such as *NCOA1* and *SDHB* are identified as being associated with cancer susceptibility. The framework further reveals distinct patterns in energy metabolism, oxidative stress, and pH regulation between cancer-prone and non-cancer-prone inflammatory diseases. These insights enhance our understanding of inflammation-associated tumorigenesis and contribute to the identification of potential biomarkers and therapeutic targets.

## 1. Introduction

Chronic inflammation represents a significant challenge to global health, imposing substantial physical and psychological burdens on patients while dramatically increasing societal healthcare costs. Its impact extends across a broad spectrum of diseases, and its intricate relationship with cancer progression has garnered considerable attention. Chronic inflammation plays a pivotal role in nearly every stage of carcinogenesis, from precancerous lesions to malignant transformation, thereby acting as a critical modulator of cancer development [1,2,3]. However, not all chronic inflammatory diseases exhibit a clear link to cancer [4,5]. This raises a fundamental question: why do certain types of chronic inflammation lead to cancer, while others remain relatively stable? At present, there is still a lack of a unified standard definition or clear biological mechanism explanation. Therefore, understanding the molecular basis behind these differences, especially identifying key genes, is essential in shedding light on why some chronic inflammations are more likely to promote cancer while others do not. Notably, these key factors may provide early prevention and diagnosis and offer potential targets for the development of novel therapeutic strategies.

Recent studies have uncovered a prevalent phenomenon of reduced protein mobility in various chronic diseases, a condition termed proteolethargy, where pathogenic signaling suppresses the mobility of proteins essential for cellular functions [6,7,8,9]. These proteins often exhibit functional dysregulation closely tied to the pathological features of chronic diseases. The research revealed that this reduction in protein mobility is strongly associated with dysregulated redox environments, highlighting a potential connection to the oxidative stress commonly seen in chronic conditions [6]. Previous studies have also established that reactive oxygen species (ROS) play a critical role in the progression of chronic diseases and cancer, with iron-mediated redox reactions such as the Fenton reaction identified as central players in these processes [10,11,12,13]. Building on these findings, this study leverages the Fenton reaction as a molecular mechanisms basis to explore the differences between cancer-prone chronic inflammatory diseases (CP-CIDs) and non-cancer-prone chronic inflammatory diseases (NCP-CIDs).

In recent years, artificial intelligence, especially deep learning, has made remarkable progress in the field of biomedicine, and has been widely used in protein structure prediction, gene expression analysis of single-cell, gene–disease association prediction, cancer subtyping, and so on [14,15,16,17,18]. Beyond capturing complex nonlinear relationships between inputs and outputs, deep learning models have also been employed as foundation models for biological reasoning and knowledge discovery [19,20,21,22,23,24,25,26]. Despite these advancements, the application of deep learning to model the cancer propensity of chronic inflammation remains underexplored. Existing studies lack both a predictive framework tailored to this problem and an approach to mining key genes and discovering new knowledge from predictive models.

In this study, we proposed FR-BINN, a novel framework for disease category prediction and interpretability analysis based on the biological knowledge-informed neural networks. In summary, the main contributions of FR-BINN are as follows:

- We established a formal definition of whether chronic inflammatory diseases are susceptible to carcinogenesis and compile transcriptomic datasets from chronic inflammation diseases to provide a foundation for predictive modeling.
- Based on the biological domain knowledge of the FR, we constructed the hierarchical knowledge neural network. The interpretable approaches were utilized to explore the important genes and the potential patterns of FR. Leveraging chain-of-thought reasoning, the large language model offers auxiliary semantic analysis and explanations.
- Extensive experiments and downstream analyses demonstrate the ability of FR-BINN, providing novel insights into the mechanisms of inflammation-driven carcinogenesis and offering potential targets for prevention and therapy.

## 2. Results

### 2.1. Overview of FR-BINN Framework

**Figure 1:**
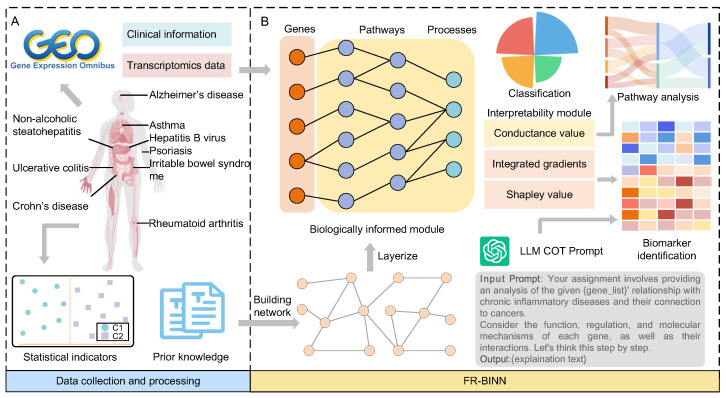
Overview of FR-BINN framework. (**A**) Integration of transcriptomic datasets, disease category definitions (derived from statistical indicators), and FR-associated biological prior knowledge. (**B**) Biologically informed neural network encoding hierarchical FR-related knowledge for classification. The framework further provides multi-level explanations (e.g., key genes, pathways) and utilizes a LLM for semantic reasoning and interpretation.

The FR-BINN framework, as illustrated in Figure 1, presents an integrated framework for prediction and interpretability analysis aimed at identifying key genes involved in the predisposition to transition from chronic inflammation to cancer. This framework consists of three core components: the biologically informed module, the interpretability module, and the large language model (LLM)-based semantic reasoning module. These modules collectively enable both key genes and biological insight extraction, ensuring that the results are reliable and interpretable.

First, we establish formal definitions for CP-CIDs and NCP-CIDs based on statistical indicators. Based on these definitions, we collected a comprehensive dataset encompassing transcriptomic profiles from diseases and control groups. Then, the biological prior knowledge, specifically gene sets and pathway interactions related to the FR, were further compiled (Figure 1A).

To mitigate shortcut learning by the model and enhance its interpretability, we incorporated the biological hierarchy knowledge into a sparse masked neural network for biologically informed module (Figure 1B). This module leverages gene sets and pathway relationships related to the FR to construct a biologically informed architecture. The resulting sparse biological hierarchy network is trained to classify samples into four distinct categories: cancer, CP-CIDs, NCP-CIDs, and normal.

Additionally, an interpretability module is developed to provide multi-level explanations, encompassing features, pathways, and higher-level biological patterns. This module plays a crucial role in identifying key genes that drive the distinction between CP-CIDs and NCP-CIDs. Furthermore, it enables the exploration of the learned patterns related to the FR.

Finally, the large language model module, built on chain-of-thought (COT) reasoning, facilitates the semantic-level interpretation of the attribution results. By harnessing the reasoning and knowledge capabilities of the LLM, this module contextualizes key findings in terms of their biological significance, linking gene attribution to inflammation-to-cancer transitions and providing an additional semantic explanation.

### 2.2. Performance Evaluation

**Figure 2:**
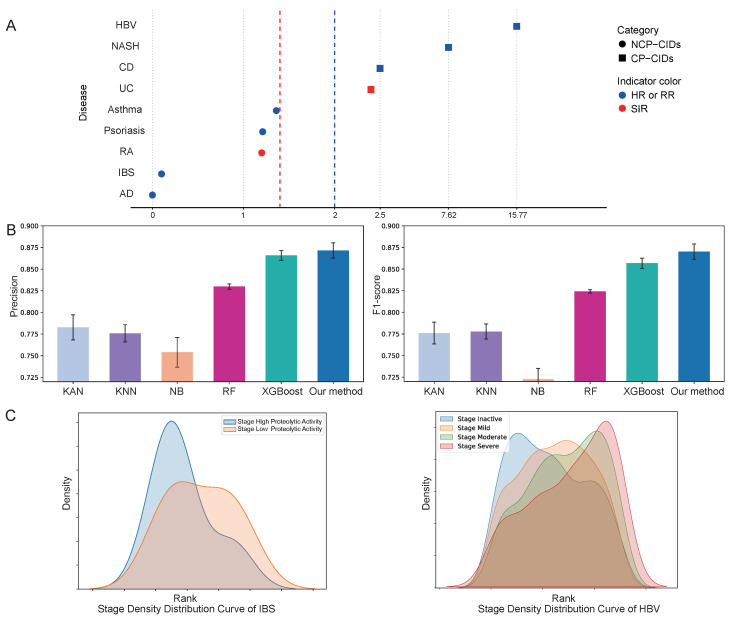
Performance evaluation. (**A**) Disease categorization into CP-CIDs and NCP-CIDs based on epidemiological statistical thresholds. (**B**) Comparative classification performance of FR-BINN versus five baseline models across categories. (**C**) Density distributions of model-predicted probabilities aligned with clinical disease stages.

Epidemiological evidence indicates that certain chronic inflammatory conditions are more likely to undergo malignant transformation, whereas others exhibit a lower propensity for cancer progression. To train and evaluate the model’s performance, we constructed a dataset and defined class labels as described in the Methods section. Chronic inflammatory diseases were categorized into CP-CIDs or NCP-CIDs based on statistical thresholds including Relative Risk (RR), Hazard Ratio (HR), and Standardized Incidence Ratio (SIR) (Figure 2A).

The biologically informed hierarchical neural network, built using prior knowledge-based network, serves as the foundation for identifying key genes associated with the cancer susceptibility of chronic inflammation diseases. To assess the predictive performance of FR-BINN in disease classification, we compared it against five widely-used machine learning algorithms: Kolmogorov–Arnold Networks (KAN) [27], k-Nearest Neighbors (KNN), Naive Bayes (NB), Random Forest (RF), and XGBoost. As shown in Figure 2B, model performance was evaluated using two key metrics: Precision and F1-score, with Recall results provided in Supplementary Figure S1. The results demonstrate that our proposed method outperforms all baselines across these metrics, achieving a Precision of 0.8715, Recall of 0.8737, and F1-score of 0.8702. These findings highlight the superior capability of the biologically informed architecture based on the FR priors, effectively distinguishing between cancer, CP-CIDs, NCP-CIDs, and normal bulk tissue samples. In addition, we conducted a comprehensive hyperparameter search to optimize model performance. Using the F1-score as the evaluation criterion, we determined the optimal settings for key hyperparameters.

Furthermore, Figure 2C depicts the density distribution of samples across different clinical stages, sorted based on predicted probabilities generated by the model. By ranking the samples and computing their density distributions, we observed the concordance between the predicted probabilities and the progression of clinical stages for specific diseases. For example, the distribution of samples for non-cancer-prone inflammatory bowel syndrome (IBS) and cancer-prone Hepatitis B virus (HBV)-associated chronic inflammation aligned closely with the observed clinical progression of these diseases. This demonstrates that beyond achieving high predictive accuracy, the model’s probability scores can provide insights into the progression dynamics of diseases.

These results collectively validate the effectiveness of FR-BINN in predicting the cancer propensity of chronic inflammation. The strong performance across classification tasks underscores the utility of integrating biological priors, while the model’s ability to capture clinically relevant patterns further reinforces its potential to uncover key genes driving inflammation-to-cancer transitions. This provides a solid foundation for subsequent interpretability, bioinformatics analysis at the gene level, and mechanism discovery at the pathway level.

### 2.3. Validation of Attribution Methods

**Figure 3:**
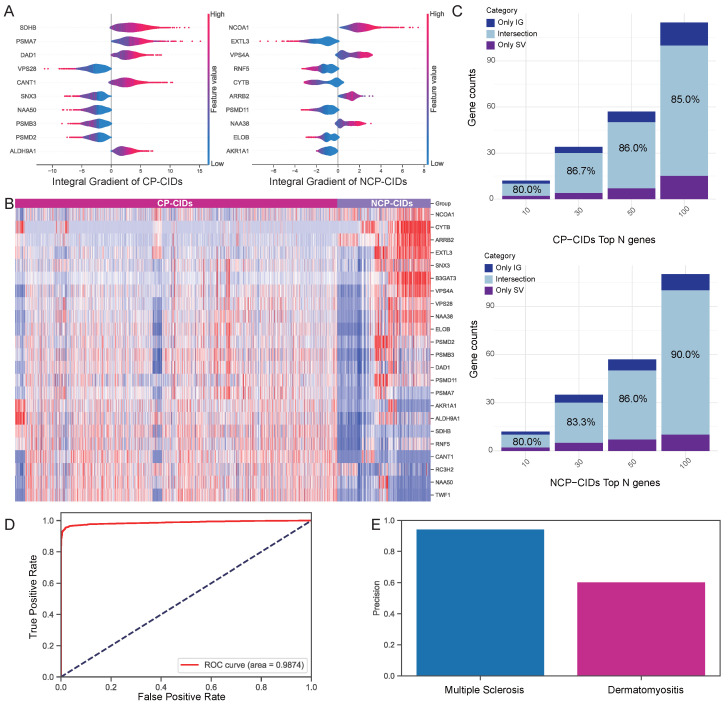
Validation of attribution methods. (**A**) Top attributed gene identification: Top 10 genes identified by the IG method that distinguish CP-CIDs from NCP-CIDs. (**B**) Expression validation: Gene expression heatmap illustrating the discriminatory power of the union of the top 10 genes identified by both IG and SV methods for cancer-prone versus non-cancer-prone inflammatory diseases. (**C**) Attribution method robustness: High concordance between IG and SV attribution results for top-ranked genes across both two inflammatory disease categories, indicating the robustness of the identified features. (**D**) Performance of the logistic regression model with refined genes. (**E**) Predictive accuracy on independent datasets.

The robustness of our framework’s gene attribution results were further validated through a combination of complementary strategies, including a visualization of the top attributed genes and gene expression heatmaps, an assessment of inter- and intra-interpretability method agreement, LLM-driven knowledge refinement, and independent classification (Figure 3).

To investigate the key genes learned by the model under the influence of biological priors, we employed the Integrated Gradients (IG) and Shapley value (SV) methods within the interpretability module to derive attribution results for both CP-CIDs and NCP-CIDs (Supplementary File S2). Figure 3A highlights the top 10 genes attributed to CP-CIDs and NCP-CIDs categories based on the IG method. For instance, genes such as *SDHB* contributed positively to the classification of CP-CIDs samples, and *NCOA1* provided evidence favoring NCP-CIDs classification.

To illustrate attribution results at the gene expression level, we visualized the union of the top 10 genes identified by both the IG and SV methods for each category in a heatmap (Figure 3B). The results reveal that the genes identified by both methods exhibit strong discriminatory power between CP-CIDs and NCP-CIDs, reinforcing the biological relevance of the attributions.

Considering that combining multiple attribution methods can reduce bias and increase robustness [28], we further assessed the overlap between the two methods within each category. Figure 3C demonstrates a high degree of overlap between the IG and SV attribution results, with intersections exceeding 80% across the top 10, 30, 50, and 100 genes for both CP-CIDs and NCP-CIDs categories. This high overlap further supports the reliability of the attribution results and suggests that the key genes identified are robust across interpretability methods. Importantly, we further explored the extent to which the same interpretability approach overlaps under both categories (Supplementary Figures S2 and S3). There is less than 20% overlap in the top 100 attribution results between CP-CIDs and NCP-CIDs categories, underscoring the distinct features that differentiate cancer-prone from non-cancer-prone inflammatory diseases. This distinction provides a valuable foundation for further exploration of the molecular mechanisms driving the differences between these two categories.

Moreover, by leveraging the LLM combined with COT, we further refined the top 10 ranked genes in both attribution methods. This was achieved by filtering out genes with weak associations to cancer progression, as determined through LLM-driven semantic explanation (Supplementary File S2). The step ensured that the remaining genes were more strongly linked to the biological processes relevant to the cancer-prone phenotype. Then, we built a logistic regression model using the refined gene sets to evaluate the model’s ability to classify samples as CP-CIDs or NCP-CIDs (Figure 3D). Finally, we tested the logistic regression model on independent disease datasets to assess its generalizability. As shown in Figure 3E, the model achieved high predictive accuracy in independent datasets for diseases such as Multiple Sclerosis in the NCP-CIDs and Dermatomyositis in the CP-CIDs, demonstrating the robustness of the attribution results across diverse contexts.

In summary, these results confirm the effectiveness of FR-BINN interpretability framework in identifying key genes that distinguish cancer-prone from non-cancer-prone chronic inflammatory diseases. The strong consistency between two attribution methods, alongside the heterogeneity of across different categories, coupled with successful validation on independent datasets, underscores the biological significance of the identified genes.

### 2.4. Analysis of Attribution Results

Following the validation of attribution methods, we computed total attribution scores for further ranking genes, investigating potential causal associations between the genes and chronic disease or cancer outcomes, and systematically analyzing the top-ranked genes.

**Figure 4:**
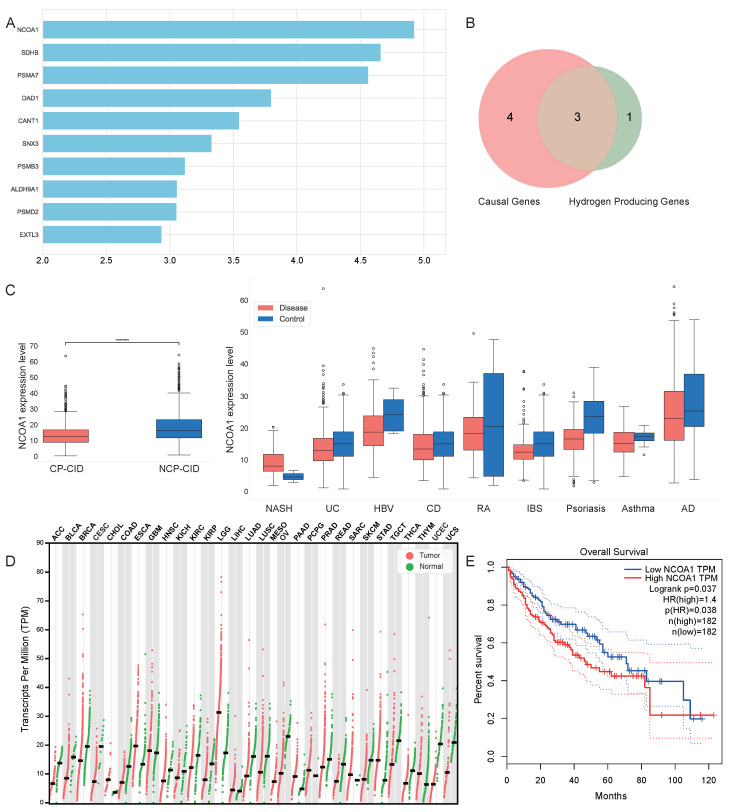
Analysis of key attributed genes. (**A**) Top 10 candidate genes ranked by combined IG and SV attribution scores. (**B**) Venn diagram illustrating, in the top 10 candidate genes, the overlap between 7 causal genes and 4 genes encoding proteins related to hydrogen ion production. (**C**) Gene expression level of *NCOA1* in CP-CIDs, NCP-CIDs, diseases, and control (********** denotes *p* = $1.47\times 10^{-32}$). (**D**) *NCOA1* expression profiles across various cancer types of TCGA. (**E**) Kaplan–Meier survival analysis for *NCOA1* in LIHC.

At first, we calculated the total attribution scores of the two feature attribution methods in CP-CIDs and NCP-CIDs. The genes were ranked based on the total attribution scores and the results of the LLM reasoning module, with the top 10 candidates visualized in Figure 4A. Among these, *NCOA1* achieved the highest total attribution score, indicating its significant contribution to the model’s classification decisions.

Using a two-sample Mendelian randomization database, we further assessed the causal relationships of the top 10 genes. Out of the top 10 genes, 7 genes (*NCOA1*, *SDHB*, *DAD1*, *SNX3*, *ALDH9A1*, *PSMD2*, and *EXTL3*) were identified as causal genes that exhibited causal relationships with diseases (Supplementary File S2). Moreover, 4 genes (*NCOA1*, *CANT1*, *ALDH9A1*, and *EXTL3*) from the top 10 list encode proteins known to catalyze hydrogen ion production reactions. The Venn diagram comparing the aboved causal genes and hydrogen production-related genes is shown in Figure 4B. Among the intersection of these two categories, three genes—*NCOA1*, *ALDH9A1*, and *EXTL3*—were identified as both causal genes and hydrogen-producing genes. This overlap implicates the potential functional importance of these genes in chronic inflammation and carcinogenesis.

Focusing on *NCOA1*, which had the highest total attribution score, we conducted a detailed expression analysis. Figure 4C illustrates the differential expression of *NCOA1* between CP-CIDs and NCP-CIDs and across nine additional diseases. *NCOA1* was consistently expressed at lower levels in disease groups compared to controls, and its expression in CP-CIDs was significantly lower than in NCP-CIDs. Further, we investigated *NCOA1* expression in cancers, as shown in Figure 4D, where its expression was found to be reduced in the majority of cancer types relative to controls. Additionally, Figure 4E illustrates a survival analysis for *NCOA1* in liver hepatocellular carcinoma (LIHC), revealing that lower *NCOA1* expression correlates with improved survival outcomes. Several studies have reported that *NCOA1* is highly expressed in various cancers and promotes tumor progression. For example, in breast cancer and prostate cancer, *NCOA1* overexpression enhances cell proliferation and metastasis [29,30]. However, it was found that the downregulation of *NCOA1* decreased cell invasion in HCC in vivo and was associated with a better 5-year survival rate in HCC patients [31]. The researchers demonstrated the pivotal role of *NCOA1* as a critical modulator in HCC metastasis, presenting a potential therapeutic target for HCC intervention. Functionally, *NCOA1* acts as a transcriptional coactivator by directly binding to transcription factors and recruiting to target gene promoters, thereby enhancing gene transcription through chromatin remodeling and transcriptional complex formation. Its emerging multiorgan oncogenic role is under intense investigation [32,33]. Mechanistically, *NCOA1* in colorectal cancer has been shown to activate JAK-STAT signaling by inhibiting SOCS1 expression and coactivate STAT3 and IRF1 to enhance Programmed death-ligand 1 (PD-L1) transcription, as well as stabilize PD-L1 protein by inhibiting SPOP-mediated proteasomal degradation, highlighting its potential as a significant biomarker across multiple tumor types [34].

Furthermore, *SDHB*, ranking second in total attribution scores, is considered a significant contributor to the model’s classification of CP-CIDs samples. *SDHB* plays a critical role in cellular and tissue metabolism by ensuring the stability of mitochondrial complexes and the proper functioning of the TCA cycle. Its proper function is crucial for mitochondrial ATP production, providing energy to cells across various tissues. Importantly, *SDHB* is linked to tumor formation [35,36]. Given its critical function in cellular metabolism and its established link to tumor development, *SDHB* is increasingly recognized as both a significant prognostic biomarker and a promising therapeutic target. Research has demonstrated that changes in *SDHB* expression can serve as a prognostic indicator in cancers such as colorectal cancer [37] and clear cell renal cell carcinoma [38]. Beyond its prognostic value, *SDHB* deficiency has been shown to be a key driver in the development of specific tumor types, including pheochromocytomas and paragangliomas [39]. In these tumors, multi-omic analyses have identified unique molecular profiles associated with metastasis, highlighting *SDHB* as a crucial target for treatment [39]. Additionally, the metabolic dysregulation caused by altered *SDHB* function, such as in hepatoblastoma, can be targeted to inhibit tumor cell proliferation [40]. The clinical utility of *SDHB* extends to its metabolic product, succinate, which can be measured as a serum biomarker for *SDHB*-mutated tumors [41], further solidifying its importance in both diagnosis and therapeutic strategies.

### 2.5. Pathway Enrichment Analysis

**Figure 5:**
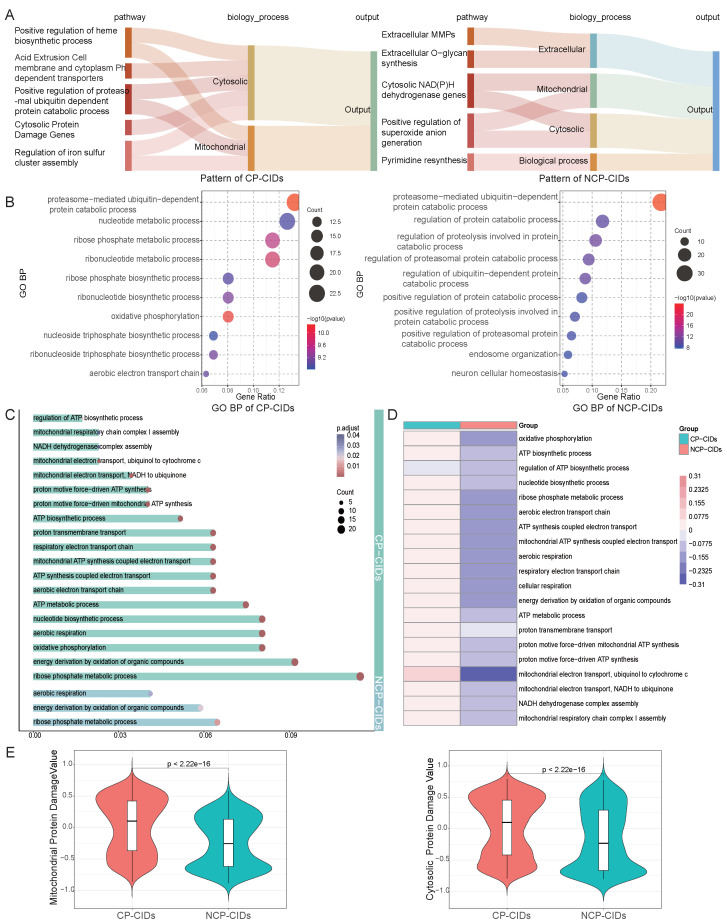
Pathway-level analysis. (**A**) Patterns in CP-CIDs and NCP-CIDs. (**B**) GO enrichment analysis. (**C**) Energy metabolism pathway enrichment results of two categories. (**D**) GSVA scores of the energy metabolism pathways. (**E**) Mitochondrial and cytosolic protein damage.

Conductance analysis reveals distinct utilization patterns of the FR between CP-CIDs and NCP-CIDs, with the top five pathways based on conductance values for both categories being presented (Figure 5A). Specifically, the model relies on divergent patterns to distinguish between disease types. These differences prompted further investigation into the underlying causes of these variations. Consequently, we explored the Gene Ontology (GO)-enriched pathways associated with the attribution results of two categories, aiming to understand the biological distinctions between these two groups at the pathway level. The full results of the pathway enrichment analysis are provided in Supplementary File S2.

The top 10 enriched pathways of biological process for CP-CIDs and NCP-CIDs are shown in Figure 5B. Compared with the pathway of NCP-CIDs, the major pathways of CP-CIDs enrichment are related to energy metabolism, including oxidative phosphorylation and the aerobic electron transport chain. Energy metabolism not only serves as the foundation for rapid cellular proliferation but also plays a pivotal role in responding to persistent microenvironmental stress. To better understand this phenomenon, we examined the role of energy metabolism in both disease categories and its potential implications.

The enriched energy metabolism pathways identified in the attribution results for both categories are illustrated in Figure 5C. CP-CIDs showed enrichment for numerous energy metabolism-related pathways, while NCP-CIDs exhibited far fewer enriched energy pathways. This observation suggests that CP-CIDs have a significantly higher energy demand. By calculating the Gene Set Variation Analysis (GSVA) scores for each pathway (Figure 5D), we found that CP-CIDs demonstrated markedly higher energy demand compared to NCP-CIDs [42]. This elevated energy requirement likely supports the rapid proliferation observed in CP-CIDs and provides essential substrates for subsequent nucleotide biosynthesis.

The choice of energy metabolism pathways not only determines cellular energy supply but also directly impacts the generation of ROS [43,44]. To further explore this, we analyzed pathways related to oxidative stress and endoplasmic reticulum stress (Supplementary Figure S4). While both categories exhibited enrichment for oxidative stress pathways, CP-CIDs also showed significant enrichment for pathways involved in hydrogen peroxide metabolism and cellular responses to free radicals. These results indicate that CP-CIDs likely face higher levels of oxidative stress, which could be a key driver of tumorigenesis. Conversely, NCP-CIDs appear to experience more controlled oxidative conditions, potentially mitigating the risk of carcinogenesis. The GSVA scores for related pathways further confirmed the heightened oxidative stress in CP-CIDs (Supplementary Figure S5).

Excess iron accumulation is a major driver of the FR, which generates hydroxyl radicals and other ROS, leading to oxidative stress, pH dysregulation, and cellular damage [45,46,47]. These effects increase the risk of cancer by causing DNA damage, protein dysfunction, and cellular instability. The pathway analysis of iron metabolism revealed that both disease categories showed enrichment for pathways related to intracellular iron ion homeostasis, highlighting the importance of maintaining iron levels in these conditions. However, CP-CIDs also exhibited enrichment for pathways related to iron–sulfur cluster assembly, suggesting that CP-CIDs experience more severe oxidative stress and require additional mechanisms to manage iron-related challenges. In contrast, NCP-CIDs appear to better maintain normal iron levels (Supplementary Figure S6). The GSVA scores for iron metabolism pathways further support these observations (Supplementary Figure S7).

In light of the observed pH disequilibrium between the intracellular and extracellular pH in various cancers, we investigated the pH regulation pathways in CP-CIDs and NCP-CIDs [48,49]. Both categories showed enrichment for pathways related to the regulation of cellular pH, macroautophagy, and intracellular pH reduction, suggesting that cells may enhance proton production to counteract the alkaline pH pressure caused by chronic inflammation and localized iron overload (Supplementary Figures S8 and S9). Notably, CP-CIDs were enriched for a wide range of nucleotide biosynthesis and catabolic metabolism pathways, particularly purine nucleotide synthesis pathways (Supplementary Figures S10 and S11). The proton-generating property of purine synthesis, coupled with reduced proton consumption during its catabolism, may serve as a compensatory mechanism to mitigate intracellular alkalinity caused by chronic inflammation and persistent Fenton reactions [50]. This mechanism could provide CP-CIDs with a means to manage persistent intracellular pH stress while simultaneously supporting rapid proliferation and biosynthetic needs [13].

Finally, we examined the extent of mitochondrial and cytoplasmic protein damage in the two categories, as shown in Figure 5E. CP-CIDs exhibited significantly higher FR intensity, reflecting the elevated oxidative stress challenges faced by this category. This heightened FR activity is likely associated with greater mitochondrial dysfunction, which could serve as a critical driver of cancer progression. In contrast, NCP-CIDs displayed lower levels of mitochondrial damage, suggesting a more stable cellular environment less prone to oncogenic transformation.

These findings underscore the significant biological distinctions between cancer-prone and non-cancer-prone chronic inflammatory diseases. CP-CIDs demonstrates higher energy demands, elevated oxidative stress, disrupted iron metabolism, and greater mitochondrial dysfunction, all of which are hallmarks of tumorigenic processes. The enrichment of purine nucleotide biosynthesis pathways in CP-CIDs further highlights a potential compensatory mechanism for pH regulation under chronic stress conditions. In contrast, NCP-CIDs exhibits a more stable metabolic and oxidative profile, which may contribute to its reduced cancer risk.

## 3. Discussion

Understanding the key factors that determine whether chronic inflammation is prone to carcinogenesis is essential to uncover the mechanisms underlying the transition from chronic inflammation to cancer. This study presents FR-BINN, a biologically informed and interpretable framework for understanding the differential cancer susceptibility of chronic inflammatory diseases. By integrating biological priors of Fenton reaction pathways with multi-layered explainable AI, our study achieves robust disease classification and uncovers critical molecular features, including key genes and pathways, through interpretability analysis. High consistency was observed in top-ranked features of the same disease category between the Integrated Gradients and Shapley values methods, while the limited overlap of the intra-interpretability method across disease categories confirmed the heterogeneity of feature attribution. The chain-of-thought-augmented large language model further filtered out biologically implausible candidates and provided semantic explanation. The refined genes achieved better predictive performance on both the chronic inflammatory disease dataset and the independent disease dataset, further validating the reliability of the attribution results.

The pathway enrichment analysis revealed key biological distinctions between cancer-prone chronic inflammatory diseases and non-cancer-prone chronic inflammatory diseases. CP-CIDs demonstrated significant enrichment in energy metabolism pathways, particularly oxidative phosphorylation and the aerobic electron transport chain, indicating heightened energy demands likely supporting rapid cellular proliferation. This elevated energy requirement, coupled with increased oxidative stress, suggests that CP-CIDs face greater challenges related to reactive oxygen species and pH regulation, which are critical drivers of tumorigenesis. In contrast, NCP-CIDs exhibited a more stable oxidative and metabolic profile, with fewer enriched energy pathways and lower oxidative stress, potentially contributing to their reduced cancer risk. Notably, CP-CIDs also showed enrichment in iron metabolism pathways, particularly those related to iron–sulfur cluster assembly, further emphasizing the severity of oxidative stress in this category. Additionally, the enriched purine nucleotide biosynthesis pathways in CP-CIDs highlight a potential compensatory mechanism to manage intracellular pH stress. These findings underscore the complex and imbalanced nature of CP-CIDs, where energy and oxidative stress imbalances may drive carcinogenesis, while NCP-CIDs appear to maintain a dynamic equilibrium that mitigates cancer risk. Extending this approach to new domains requires considering the biological plausibility between pathway relationships and disease classification. Future work may extend the framework to broader disease contexts and incorporate additional omics data to further enhance its predictive and interpretative capabilities.

In summary, this study establishes a robust framework that combines AI-driven modeling with biological insights to tackle the long-standing challenge of understanding the cancer propensity of chronic inflammation. By bridging the gap between predictive accuracy and interpretability, FR-BINN provides valuable insights into inflammation-cancer transitions and suggests potential avenues for the development of novel diagnostic and therapeutic strategies.

## 4. Materials and Methods

### 4.1. Datasets and Data Processing

In this study, we curated a comprehensive dataset comprising 4284 transcriptomic samples sourced from the Gene Expression Omnibus (GEO) [51]. These samples represent four primary categories: cancer-prone chronic inflammatory diseases (CP-CIDs), non-cancer-prone chronic inflammatory diseases (NCP-CIDs), cancer, and normal samples. Notably, the normal samples were derived from the control cohorts of NCP-CIDs and CP-CIDs studies. A total of 12 diseases were included in the analysis, spanning a diverse range of conditions: Asthma, Alzheimer’s disease (AD), Psoriasis, Irritable bowel syndrome (IBS), Rheumatoid arthritis (RA), Ulcerative colitis (UC), Crohn’s disease (CD), Non-alcoholic steatohepatitis (NASH), Hepatitis B virus (HBV), Colon cancer, Colorectal cancer, and Hepatocellular carcinoma (HCC). Detailed information on the GEO dataset accession numbers is provided in Supplementary Tables S1 and S2. Additionally, two independent datasets, including multiple sclerosis and dermatomyositis, were retrieved from GEO to serve as external validation sets (Supplementary Tables S3 and S4).

To ensure consistency and compatibility across datasets, all the transcriptome data that were used were processed through a unified pipeline established by the NCBI SRA and GEO teams (www.ncbi.nlm.nih.gov/geo/download/?acc=GEONumber, accessed on 3 June 2024). This pipeline involved the re-alignment and quantification of raw sequencing data to produce high-quality, harmonized expression profiles. The expression levels were quantified using transcripts per kilobase million, a normalization metric that facilitates cross-study comparison by accounting for both sequencing depth and gene length. This standardized preprocessing approach ensured robust data integration and reproducibility in downstream analyses. The inclusion of a larger and more diverse dataset aimed to improve the robustness and generalizability of gene-level attribution results, allowing the model to better capture class-specific biological signatures. Cancer samples were chosen to reflect malignancies associated with high inflammation-to-cancer risk, which are also the primary tissues involved in CP-CIDs. During data processing, we retained only transcriptomic samples derived from the disease-relevant tissue types to ensure biological relevance and reduce confounding effects.

### 4.2. The Definition of NCP-CIDs and CP-CIDs

Chronic inflammation has been firmly established as a key factor in the initiation and progression of tumors, with inflammatory conditions significantly increasing tumor risk. Epidemiological evidence reveals that certain chronic inflammatory conditions exhibit a higher likelihood of malignant transformation, while others appear less prone to cancer progression. However, a standardized framework to categorize these conditions is currently lacking.

Drawing from extensive literature reviews and statistical analyses of large population cohorts, we propose a quantitative definition for determining the susceptibility of chronic inflammation to carcinogenesis:

(1) prone_or_not=proneif(RR>2)∨(HR>2)1if∨(SIR>1.4)not_proneelse

where RR, HR, and SIR represent Relative Risk, Hazard Ratio, and Standardized Incidence Ratio, respectively. Based on this definition, we categorized chronic inflammatory diseases into NCP-CIDs and CP-CIDs (Supplementary Table S5). The thresholds of RR > 2 and HR > 2 align with established epidemiological conventions where an effect size exceeding 2.0 signifies a clinically important association and substantially elevated risk [52,53,54]. Our analysis of large-scale cohort studies and meta-analyses revealed that diseases consistently exhibiting RR > 2, HR > 2, or SIR > 1.4 demonstrate a clinically significant increase in cancer risk compared to reference populations. Crucially, empirical data aggregation showed clear separation: CP-CIDs consistently exceeded these thresholds, while NCP-CIDs fell below them. This natural dichotomy informed our threshold selection to robustly distinguish risk categories. These thresholds may warrant refinement if applied to broader disease spectra. Nevertheless, they provide a rigorously justified foundation for our framework’s classification task.

We first identified five diseases as NCP-CIDs: Asthma, AD, Psoriasis, IBS, and RA. Below, we summarize their characteristics and relationships with cancer risk.

Asthma: The Global Initiative for Asthma defines Asthma as a heterogeneous disease characterized by chronic airway inflammation and symptoms such as wheezing, coughing, dyspnea, and chest tightness [55]. While severe Asthma has been associated with an increased risk of certain cancers, the HR remains moderate at 1.36 [56,57].

Alzheimer’s Disease: AD is a neurodegenerative disorder marked by progressive cognitive decline and psychiatric disturbances [58]. Epidemiological studies consistently show an inverse association between AD and cancer risk. Patients with AD are significantly less likely to develop overall malignancies or specific cancers compared to the general population [4,59,60,61].

Psoriasis: Psoriasis is a chronic inflammatory disease of the skin and joints. While patients with Psoriasis have a slightly increased cancer risk, the RR remains modest at 1.21 [62].

Irritable Bowel Syndrome: IBS is a chronic gastrointestinal condition affecting 7–21% of the general population [63]. Despite its prevalence, IBS does not increase overall cancer risk. In fact, IBS is associated with a reduced risk of colorectal cancer and cancer-specific mortality [64].

Rheumatoid Arthritis: RA is a systemic autoimmune disease characterized by persistent joint inflammation and the presence of autoantibodies [65]. Patients with RA exhibit an increased overall cancer risk, with a SIR of 1.20 [66].

Next, the following diseases UC, CD, HBV, and NASH are considered to be CP-CIDs.

Inflammatory Bowel Disease: UC and CD are the primary forms of inflammatory bowel disease, distinguished by differences in genetic predisposition, clinical features, and histopathological characteristics [67]. UC increases the risk of colorectal cancer, with a pooled SIR of 2.4 and a RR of 2.4 [68]. Similarly, CD significantly elevates the risk of colorectal and small bowel cancers, with a RR of 2.5 [69].

Non-Alcoholic Steatohepatitis: NASH, a more severe form of Non-alcoholic fatty liver disease, is characterized by necroinflammation and accelerated fibrosis progression [70]. Patients with NASH are at a significantly increased risk of developing HCC, with a HR of 7.62 [71].

Hepatitis B Virus: HBV is a major risk factor for HCC. After 4.4 million person-years of follow-up, participants seropositive for Hepatitis B surface antigen exhibited a dramatically increased risk of HCC, with an HR of 15.77 [72].

Finally, we employed independent datasets for two additional disease datasets: dermatomyositis in the CP-CIDs and multiple sclerosis in the NCP-CIDs [73,74,75,76]. These datasets were not used during training, enabling robust validation of model predictions and biological insights.

### 4.3. Construction of Prior Networks

The FR is a critical biochemical process that generates hydroxyl radicals (•OH) and hydroxide ions ($OH^{-}$) via the interaction of ferrous iron ($Fe^{2+}$) and hydrogen peroxide ($H_{2}O_{2}$):

(2) $$Fe^{2+}+H_{2}O_{2}⟶Fe^{3+}+•OH+OH^{-}$$

Hydroxyl radicals, as one of the most reactive oxidative species, have been extensively documented to cause severe cellular damage, including lipid peroxidation and DNA damage, while hydroxide ions significantly influence intracellular and extracellular pH homeostasis, which is fundamental to maintaining cellular function [13,50,77,78]. The FR has been implicated in cancer and chronic inflammatory diseases [10,50]. To construct the prior network, we curated the genesets of FR and relationships of biological entities from the existing literature [50,77,79]. This network captures key pathways and molecular components associated with FR processes, including genes such as iron homeostasis, ROS production, and antioxidant defense. The prior network was structured into layered form, denoted as *S*, and integrated into the FR-BINN framework to enable biologically grounded prediction and interpretability in the context of CP-CIDs and NCP-CIDs.

### 4.4. Construction of Model

We propose FR-BINN, a framework for interpretable analysis of disease prediction based on the biological hierarchy knowledge, which can not only be used to classify cancer, NCP-CIDs, CP-CIDs, and normal controls, but can also explore multiple levels of interpretation of features and pathways (Figure 1). The framework comprises three primary modules, each designed to address specific objectives. The first biological hierarchy knowledge module incorporates prior knowledge to reduce model parameters and mitigate shortcut learning. The second is the interpretability module, which explores and analyzes feature and pathway importance across different categories, mainly using the Integral Gradient, Shapley value, and conductance. Leveraging chain-of-thought reasoning, the large language model-based semantic module refines the identification of key genes and reduces potential hallucinations, contributing to more accurate and interpretable semantic explanations.

Biologically informed module: In order to reduce the shortcut learning and improve the interpretability of the model, we first use the prior knowledge of biology, FR-related gene sets and pathway relationships, and construct a prior-based sparse neural network. This hierarchical knowledge also helps to reduce the number of parameters and improve the performance of the model [23,25,26,80]. Training with different types of data helps FR-BINN to learn how to distinguish the state of the sample by using the biological prior network and the transcript expression data of the sample. This architecture of the biology knowledge-informed module was built using the pathway of the fention reaction. In FR-BINN, each node encodes some biological entity (for example, genes and pathways) and each edge represents a known relationship between the corresponding entities. The knowledge on the edges leads to a smaller number of parameters compared to fully connected networks with the same number of nodes, and thus potentially fewer computations.

(3) $$\begin{array}{cc} \; & h_{\left(l+1\right)}=f\left({\left(S_{l}⊙W_{l}\right)}^{T}h_{l}+b\right) \end{array}$$

(4) $$\begin{array}{cc} \; & pred=softmax(W_{out}h_{out}+b) \end{array}$$

where $h_{0}=x$, and *x* represents the input feature, which, here, is the gene expression value. $S_{l}\in n^{\left(l+1\right)\times n^{\left(l\right)}}$ denotes the masked matrix of the layered prior knowledge. The activation function *f* introduces nonlinearity, while the Hadamard product ⊙ imposes sparsity by incorporating prior knowledge into the weights *W*. The model is trained using the Adam optimizer, and the cross-entropy loss function is employed to minimize classification errors:

(5) $$L=-\frac{1}{N}\sum_{i=0}^{N-1}\sum_{k=0}^{K-1}\left(y_{ik}\right)log\left(pred_{ik}\right)$$

where *N* represents the number of samples, *K* is the number of categories, and *y* is the ground truth.

Interpretability module. To identify and analyze key biological entities in a framework grounded in prior biological knowledge, we integrated Integrated Gradients (IG) [17,81], Shapley value (SV) [82], and conductance [83,84] into the interpretability module of FR-BINN. These methods enable the evaluation of feature attributions and the understanding of neuron importance in the prediction process. Together, these methods enhance the interpretability of predictions at multiple levels, from input features to network layers.

IG is an attribution method designed to quantify the contribution of each input feature to a model’s output. It works by integrating the gradients of the model’s output with respect to the input features along a straight path from a baseline input to the actual input. IG satisfies two key axioms: Sensitivity (ensuring that the attribution score reflects the influence of the feature) and Implementation Invariance (ensuring consistency across functionally equivalent models).

(6) $$IG_{i}=\left(x_{i}-x_{i}^{\prime }\right)\sum_{k=1}^{m_{ig}}\frac{\partial F(x^{\prime }+\frac{k}{m_{ig}}\left(x-x^{\prime }\right))}{\partial x_{i}}\frac{1}{m_{ig}}$$

where *F* represents our deep neural network. $IG_{i}$ signifies the final attribution score with respect to the $i_{th}$ dimension of the features. The baseline $x^{\prime }$ is set to zero. $m_{ig}$ denotes the number of steps used in the Legendre–Gauss quadrature integral approximation.

The concept of total conductance is modified from IG for computing internal neuron importance. Consider a specific neuron y in a hidden layer of a network. We can define the conductance of neuron y for the attribution to an input variable i as follows:

(7) $$Cond_{i}^{y}\left(x\right)::=\left(x_{i}-x_{i}^{\prime }\right)\cdot \int_{\alpha =0}^{1}\frac{\partial F(x^{\prime }+\alpha \left(x-x^{\prime }\right))}{y}\cdot \frac{\partial y}{\partial x_{i}}d\alpha$$

the total conductance of the hidden neuron y by summing over the input variables is defined as follows:

(8) $$Cond^{y}\left(x\right)::=\sum_{i}\left(x_{i}-x_{i}^{\prime }\right)\cdot \int_{\alpha =0}^{1}\frac{\partial F(x^{\prime }+\alpha \left(x-x^{\prime }\right))}{y}\cdot \frac{\partial y}{\partial x_{i}}d\alpha$$

Additionally, we can aggregate over a set of logically related neurons that belong to a specific hidden layer. To define the whole conductance of the set, we can sum over the conductances of the neurons in the set. As with the IG, we use an approximation algorithm [84] to compute the conductance, with a step size of $m_{con}$.

The SV, rooted in cooperative game theory, provides a principled way to fairly distribute the payoff among input features by quantifying their marginal contributions to the prediction across all possible feature combinations. While the exact computation of SVs is computationally expensive due to the combinatorial number of possible feature subsets, Kernel SHAP approximates the SVs efficiently by leveraging a weighted linear regression method inspired by the local interpretable model-agnostic explanations framework [82]. Kernel SHAP constructs a surrogate interpretable model by sampling feature subsets and calculating the original model’s predictions for those subsets, assigning weights based on their importance. Here, we use $m_{shapley}$ to represent the approximate number of times the original model is queried to generate predictions for training the surrogate interpretable model.

Large language model-based semantic reasoning module. The third module of FR-BINN leverages the LLM to analyze key genes. This module employs COT reasoning to enhance the model’s explanation, providing context-aware answers while reducing hallucinations.

Recent advancements in LLMs have demonstrated their potential in achieving near-human-level intelligence by training on vast amounts of knowledge from the web and other domains [85,86,87]. Studies have shown that COTreasoning improves LLMs’ capabilities in logical reasoning and decision-making tasks, making them more effective for complex, multi-step problem-solving scenarios [88,89]. In our framework, we utilize prompt engineering and COT reasoning to guide LLMs assistance in interpreting relationships between key genes and disease states. The standard prompting mechanism in LLMs can be represented as follows:

(9) $$p\left(A|T,Q\right)=\prod_{i=1}^{\left|A\right|}p_{\mathrm{LLM}}\left(a_{i}|T,Q,a_{<i}\right)$$

where *Q* represents the reasoning question, *T* denotes the prompt, $p_{LM}$ is the parameterized probabilistic model, and *A* is the answer. |A| represents the length of the final answer. $a_{i}$ denotes the *i*-th token. This equation aims to maximize the likelihood of answer. To incorporate COTreasoning, the equation is reformulated by introducing intermediate reasoning steps:

(10) p(A|T,Q)=p(A|T,Q,R)p(R|T,Q)

(11) $$p\left(R|T,Q\right)=\prod_{i=1}^{\left|R\right|}p_{\mathrm{LLM}}\left(r_{i}|T,Q,r_{<i}\right)$$

(12) $$p\left(A|T,Q,R\right)=\prod_{j=1}^{\left|A\right|}p_{\mathrm{LLM}}\left(a_{i}|T,Q,R,a_{<j}\right)$$

where the prompt $T={\left(Q_{i},R_{i},A_{i}\right)}^{K_{i=1}}$, $r_{i}$ is one step of total |R| reasoning steps.

### 4.5. Evaluation Metrics

In our experiment, the five-fold cross-validation was utilized for evaluating method performance. For the classification task, the folds are made by preserving the percentage of samples for each class. In addition, we use the weighted Precision, weighted Recall and the weighted F1-score to evaluate performance.

### 4.6. Implementation

We implemented a layered architecture consisting of two pathway layers and one gene layer, though users can define custom hierarchies and relationships based on their gene sets or pathways of interest. Higher layers in the hierarchy represent higher-order biological processes or pathways. The model input layer is the value of omics, which in our task is the transcriptional expression of genes. The model output layer is used for classification. Our implementation of FR-BINN is based on the PyTorch machine learning framework, version 2.3. Using the F1-score as the evaluation criterion, we determined the optimal settings for key hyperparameters: the number of epochs was found to be optimal at 500, the batch size performed best at 32, and the learning rate achieved maximum performance at 0.001. To uncover latent biological insights, we trained the model on the complete dataset after determining optimal hyperparameters for the biological prior module. To ensure robustness and biological interpretation, attribution analyses were computed exclusively using correctly predicted samples. For each sample, we focused solely on the attribution patterns corresponding to its true class label. The values of $m_{ig}$, $m_{con}$, and $m_{shapley}$ are all set to 800. The large language model used is GPT4O. In addition, the filtering process relied on the LLM’s semantic explanations generated through COT prompting. Genes were retained only if the LLM’s narrative established mechanistic relevance to cancer development. To validate the disease relevance of identified genes, we interrogated the Mendelian Disease Database (DMRdb) for evidence of established causal associations [90]. Attribution ranking scores were computed as the mean absolute values of sample-level attributions. To obtain total attribution rankings, we derived a composite score by summing the attribution values from both feature attrbution methods. Gene enrichment analysis was performed using the union of genes identified by both interpretability methods within each class. For each class, we selected the top 5% of genes of the pathways based on total attribution scores and ranking values. Significant enrichment results were defined as those with adjusted *p*-values < 0.05. GSVA scores were calculated according to [42], with group-level GSVA scores represented as the mean score across all group samples.

## 5. Conclusions

Our study introduced FR-BINN, a novel biologically informed neural network framework designed to classify chronic inflammatory diseases based on their propensity for carcinogenesis and to uncover the underlying molecular mechanisms. By integrating biological priors of the Fenton reaction with advanced interpretability methods, our work provides significant insights into the transition from inflammation to cancer. The core contributions, potential applications, and future directions of study are as follows:

- FR-BINN effectively classifies samples into four categories by integrating a hierarchical structure based on FR-related pathways. This biologically informed design enhances model performance and ensures the interpretability of its predictions.
- Through a multi-method interpretability analysis, we identified and validated key biomarkers that are critical in distinguishing CP-CIDs from NCP-CIDs. These identified key genes are promising candidate biomarkers for early diagnosis and therapeutic targets.
- Our analysis revealed clear differences in energy metabolism, oxidative stress, and pH regulation between CP-CIDs and NCP-CIDs. This understanding suggests that therapies could be developed to modulate these metabolic pathways or mitigate oxidative stress specifically in CP-CIDs, potentially preventing cancer progression.
- While FR-BINN effectively leverages the strengths of biologically informed neural networks for gene and complex pattern recognition, future research could benefit from incorporating more classical mathematical modeling approaches [91,92,93]. Although these methods typically require substantial mathematical expertise and a firm grasp of the underlying physical or biological mechanisms, they offer complementary perspectives and have the potential to enhance predictive power. This is particularly valuable for long-term predictions or when establishing precise causal links is paramount.

## Data Availability

All data utilized in this study are derived from publicly available the GEO database (www.ncbi.nlm.nih.gov/geo/, accessed on 3 June 2024). Detailed information is provided within the article.

## Supplementary Materials

The following supporting information can be downloaded at: https://www.mdpi.com/article/10.3390/ijms26146670/s1.
