# Supplementary material for: FR-BINN: Biologically Informed Neural Networks for Enhanced Biomarker Discovery and Pathway Analysis
Source: Int J Mol Sci. 2025 Jul 11;26(14):6670. doi: 10.3390/ijms26146670 (PMC12294759; doi:10.3390/ijms26146670)
Supplement: Supplementary file 1 [file ijms-26-06670-s001.zip › Supplementary File1.pdf]

1. FIGURES AND TABLES

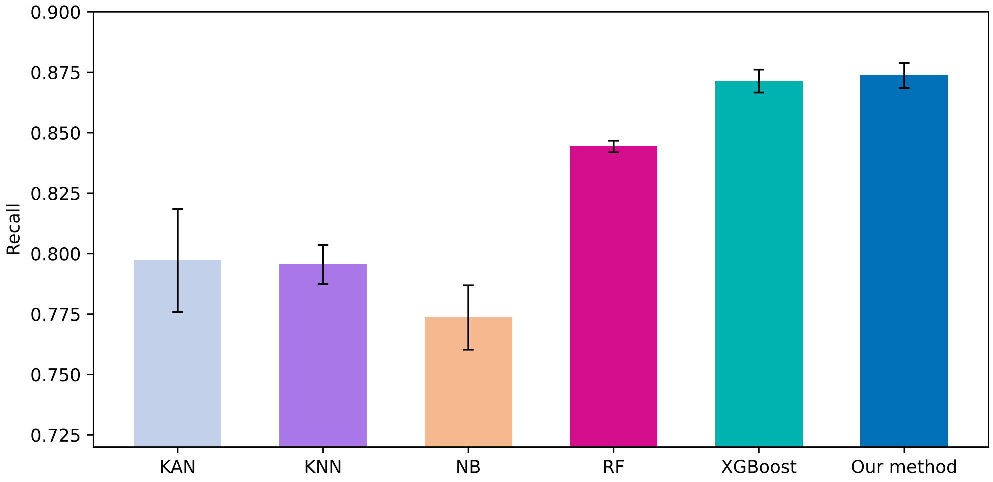

Fig. S1. Recall scores.

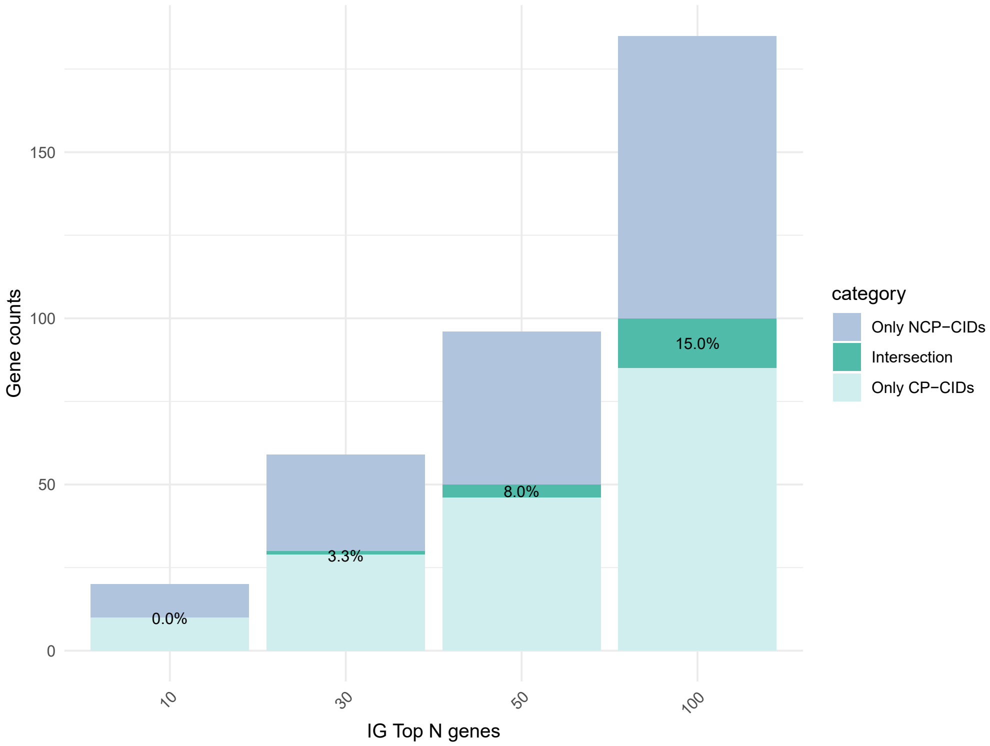

Fig. S2. Overleap of attribution results of two inflammatory categories of IG.

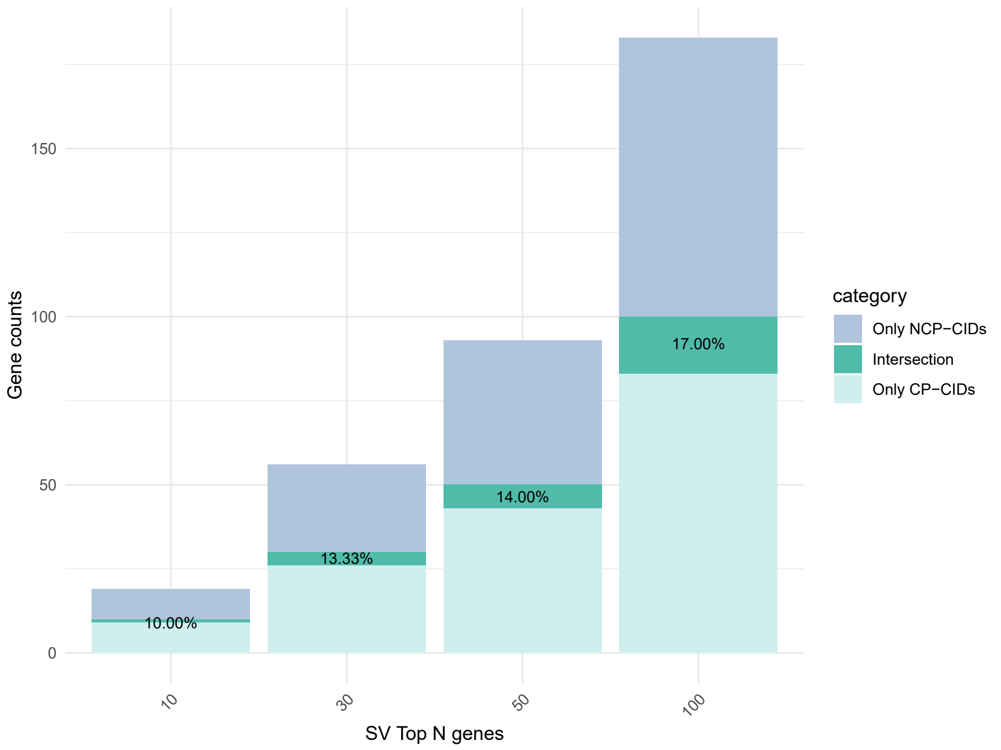

Fig. S3. Overleap of attribution results of two inflammatory categories of SV.

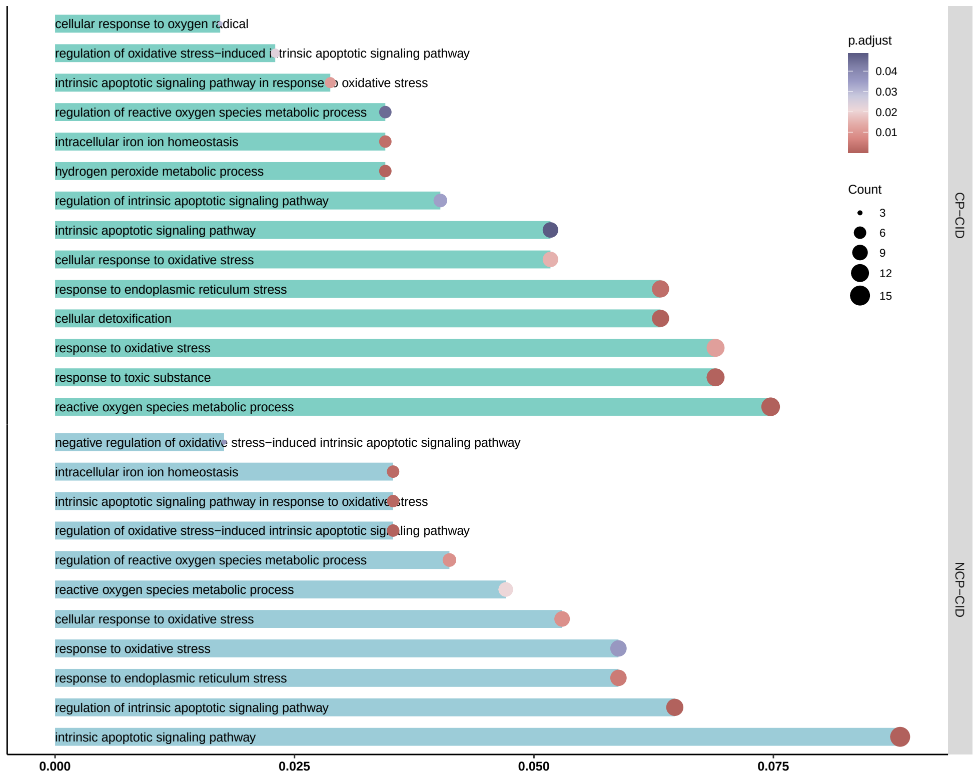

Fig. S4. GSEA of ROS.

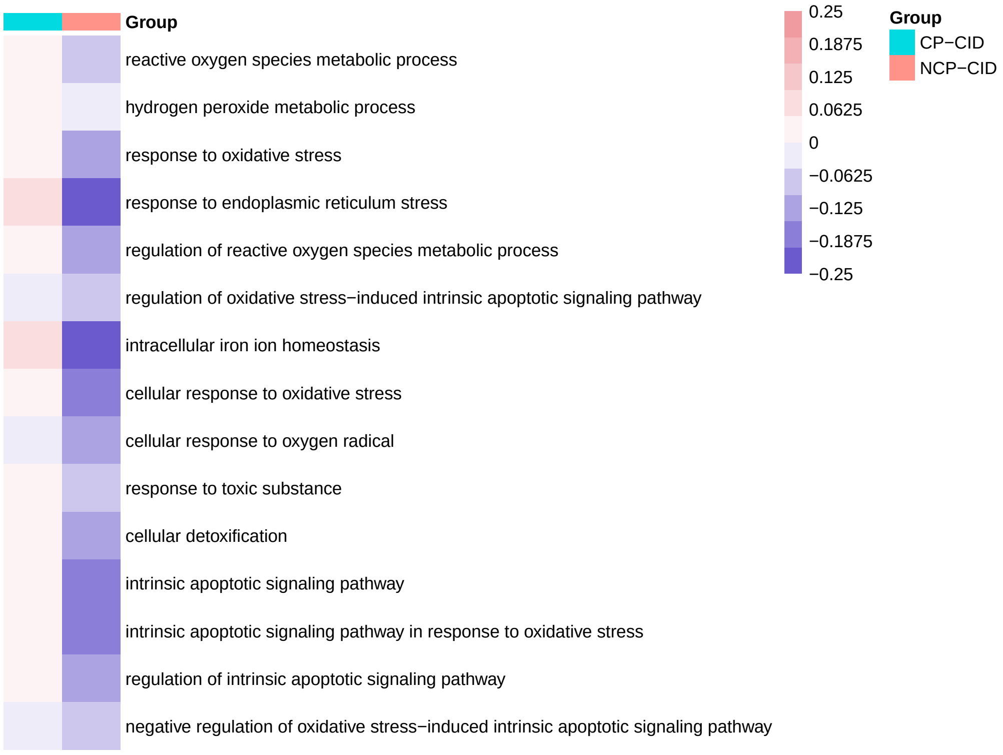

Fig. S5. GSVA of ROS.

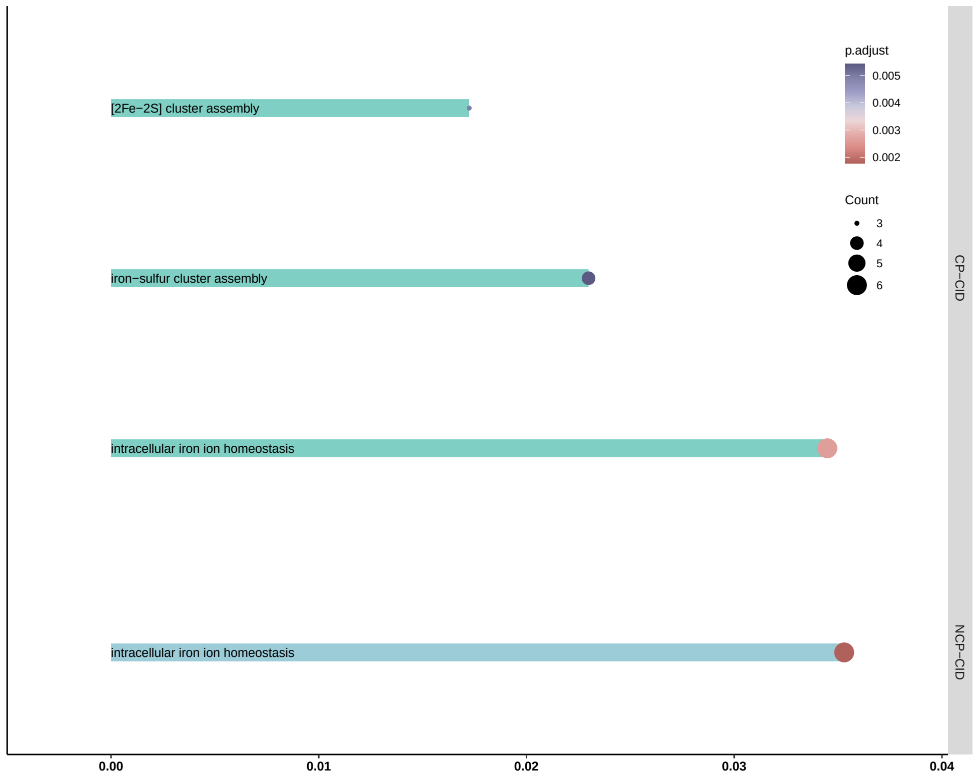

Fig. S6. GSEA of iron.

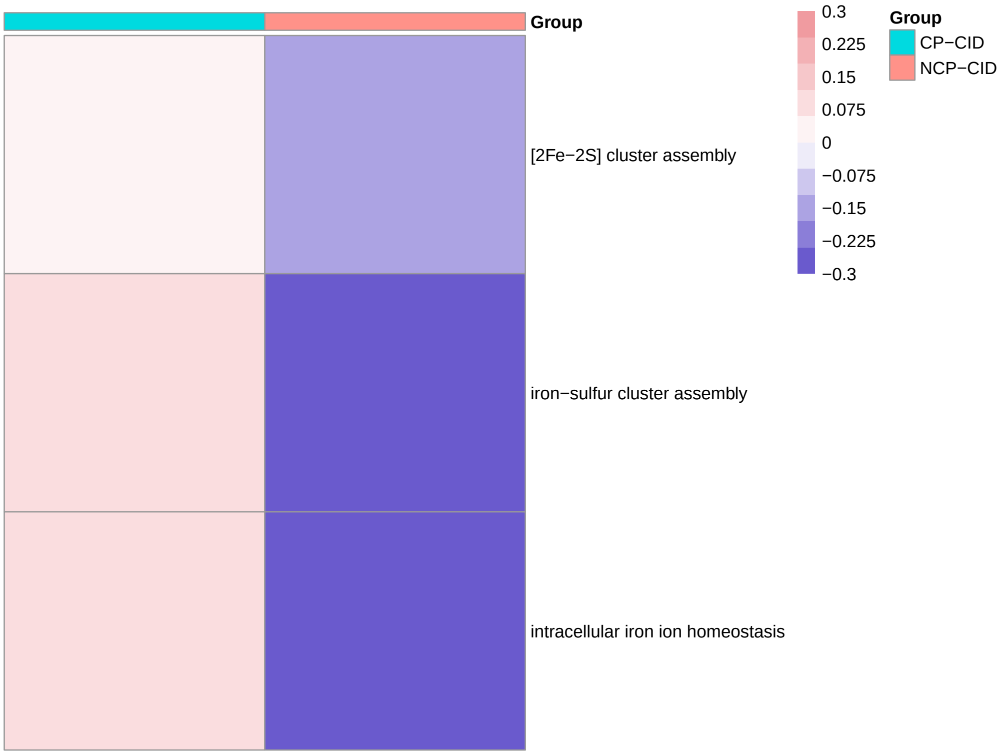

Fig. S7. GSVA of iron.

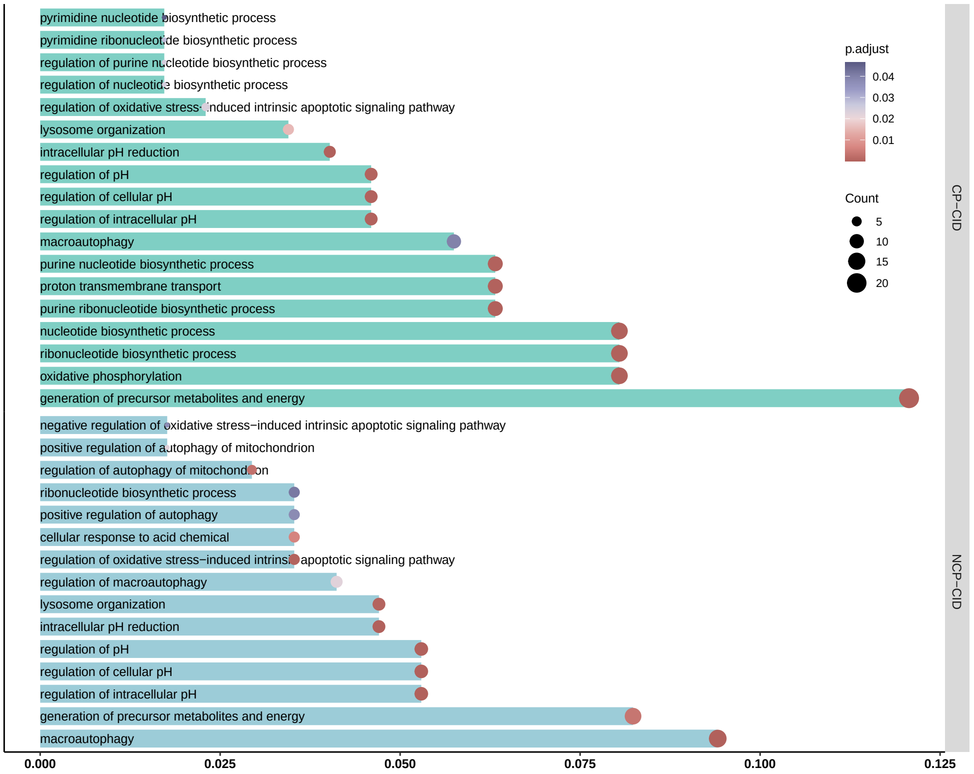

Fig. S8. GSEA of pH.

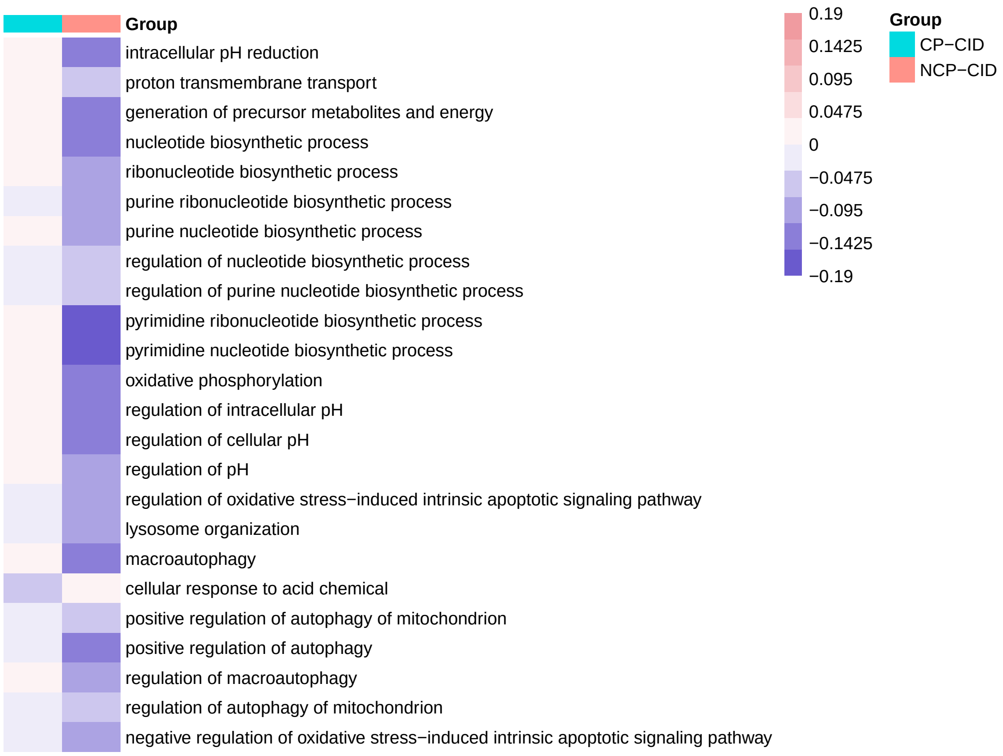

Fig. S9. GSVA of pH.

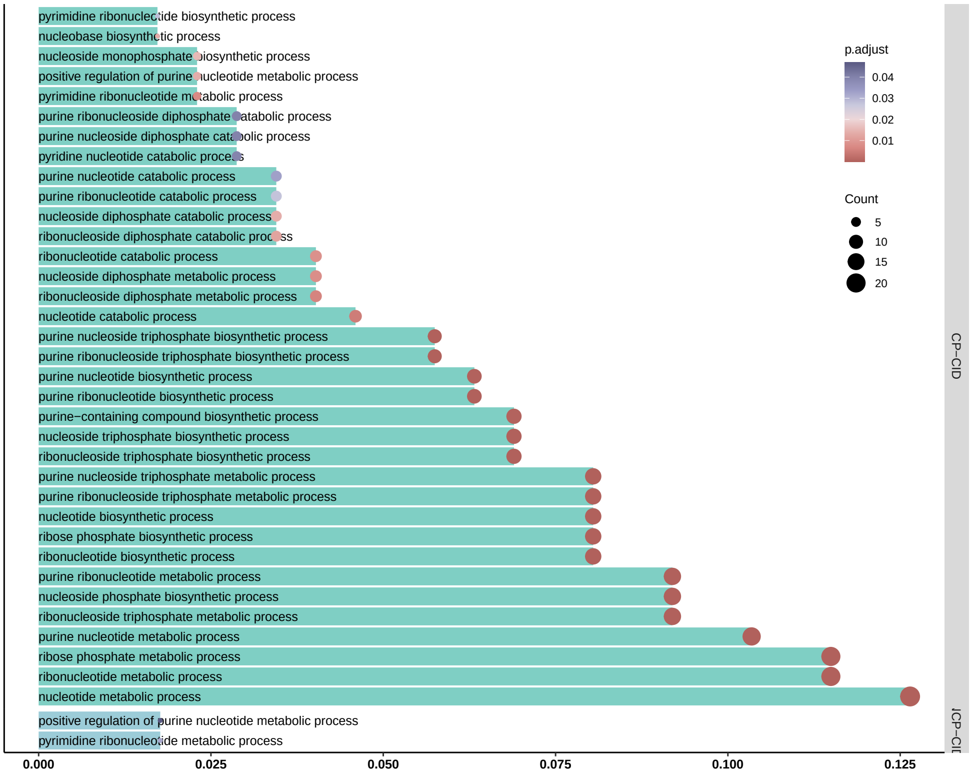

Fig. S10. GSEA of nucleotide.

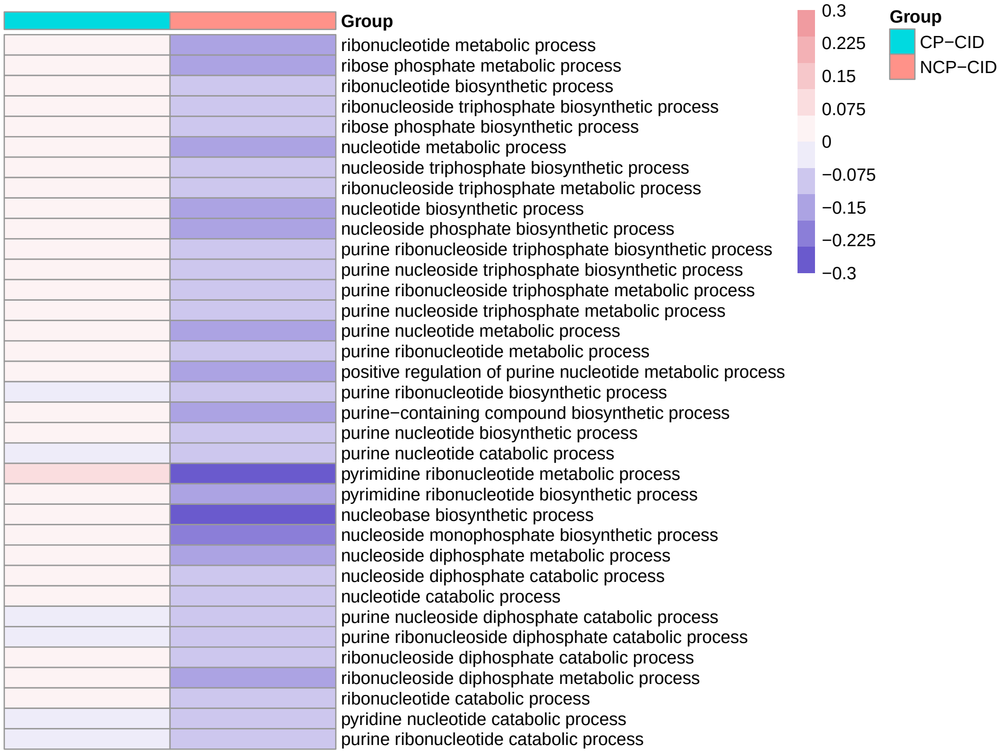

Fig. S11. GSVA of nucleotide.

Table S1. Disease categories

Disease Type	Disease Name
NCP-CIDs	Asthma
	Alzheimer's disease (AD)
	Psoriasis
	Irritable bowel syndrome (IBS)
	Rheumatoid arthritis
CP-CIDs	Ulcerative colitis (UC)
	Crohn's disease (CD)
	Inflammatory bowel disease (IBD)
	Non-alcoholic steatohepatitis (NASH)
	Hepatitis B virus (HBV)
Cancers	Colon cancer
	Colorectal cancer
	Hepatocellular carcinoma (HCC)

Table S2. GSE number of diseases

**Disease name**	**GSE Number**
Alzheimer's disease	GSE104704 GSE125583 GSE173955 GSE53697
Asthma	GSE159091 GSE85567
Psoriasis	GSE121212 GSE114286 GSE114729 GSE120795 GSE186117 GSE205748 GSE186063 GSE142582
Irritable bowel syndrome	GSE146853 GSE166869 GSE168759
Rheumatoid arthritis	GSE89408
Ulcerative colitis	GSE193677 GSE227747 GSE137344 GSE130038
Crohn's disease	GSE193677 GSE137344 GSE227747 GSE164871 GSE192786
Inflammatory bowel disease	GSE193677 GSE137344 GSE120795 GSE164871 GSE227747
Non-alcoholic steatohepatitis	GSE126848 GSE167523 GSE175448 GSE120795
Hepatitis B virus	GSE230397
Colon cancer	GSE137327 GSE33782 GSE104178
Colorectal cancer	GSE180440 GSE223119 GSE237684 GSE50760
Hepatocellular carcinoma	GSE65485 GSE94660 GSE113617 GSE198946 GSE135631 GSE195952 GSE124535

Table S3. Disease category of independent dataset

Disease Type	Disease Name
NCP-CIDs	Dermatomyositis
CP-CIDs	Multiple Sclerosis (MS)

Table S4. GSE number of independent dataset

Disease	GEO Number
Dermatomyositis DM	GSE143323
Multiple Sclerosis MS	GSE123496 GSE137619 GSE100297

Table S5. Disease ratio

Disease Type	Disease name	Ratio
NCP-CIDs	Asthma	HR 1.36
	Alzheimer′s disease	inverse association
	Psoriasis	RR 1.21
	Irritable bowel syndrome	not increase risk
	Rheumatoid arthritis	SIR 1.20
CP-CIDs	Ulcerative colitis	SIR 2.4
	Crohn′s disease	RR 2.5
	Non-alcoholic steatohepatitis	HR 7.62
	Hepatitis B virus	HR 15.77
